# Current vaccines, experimental immunization trials, and new perspectives to control selected vector borne blood parasites of veterinary importance

**DOI:** 10.3389/fvets.2024.1484787

**Published:** 2024-11-13

**Authors:** Heba F. Alzan, Mona S. Mahmoud, Carlos E. Suarez

**Affiliations:** ^1^Department of Veterinary Microbiology and Pathology, College of Veterinary Medicine, Washington State University, Pullman, WA, United States; ^2^Parasitology and Animal Diseases Department, Veterinary Research Institute, National Research Center, Giza, Egypt; ^3^Animal Disease Research Unit, Agricultural Research Service, United States Department of Agriculture, Pullman, WA, United States

**Keywords:** vaccine, farm animals, vector borne, ticks, blood sucking flies, parasites

## Abstract

Parasite infections transmitted by vectors such as ticks and blood-sucking arthropods pose a significant threat to both human and animal health worldwide and have a substantial economic impact, particularly in the context of worsening environmental conditions. These infections can manifest in a variety of symptoms, including fever, anemia, jaundice, enlarged spleen, neurological disorders, and lymphatic issues, and can have varying mortality rates. In this review, we will focus on the current state of available vaccines, vaccine research approaches, and trials for diseases caused by vector-borne blood parasites, such as *Babesia*, *Theileria*, *Anaplasma*, and *Trypanosoma*, in farm animals. Control measures for these infections primarily rely on vector control, parasiticidal drug treatments, and vaccinations for disease prevention. However, many of these approaches have limitations, such as environmental concerns associated with the use of parasiticides, acaricides, and insecticides. Additionally, while some vaccines for blood parasites are already available, they still have several drawbacks, including practicality issues, unsuitability in non-endemic areas, and concerns about spreading other infectious agents, particularly in the case of live vaccines. This article highlights recent efforts to develop vaccines for controlling blood parasites in animals. The focus is on vaccine development approaches that show promise, including those based on recombinant antigens, vectored vaccines, and live attenuated or genetically modified parasites. Despite intensive research, developing effective subunit vaccines against blood stage parasites remains a challenge. By learning from previous vaccine development efforts and using emerging technologies to define immune mechanisms of protection, appropriate adjuvants, and protective antigens, we can expand our toolkit for controlling these burdensome diseases.

## 1 Introduction

The impact of blood parasites is scaling up globally. The increasing costs of control using acaricides, insecticides, and treatments of infected animals, associated with the emergence of drug resistance and environmental hazards, accelerated efforts to find effective vaccines against hemoparasites using rational approaches. Moreover, it is known that animals that resolve and recover from acute blood-borne infectious diseases, usually become asymptomatic carriers for the pathogens and may act as reservoirs of the infective agents that cannot be clinically differentiated from the non-infected ones (1). Alternative methods for the control of hemoparasites usually involve targeting their vectors, which is frequently a very difficult approach due to the existence of wildlife reservoirs and the development of acaricide resistance, but vector control could also be achieved by developing effective anti-vector vaccines.

Despite many efforts on the development of efficient vaccines, there are still many factors that can impact the efficacy of vaccines. These combined factors may include issues associated with the hosts, human-related activities, and the types of vaccines.

Among host related factors, it should be considered: (i) animal breed variations; (ii) age-related factors; and (iii) incubation periods in case of infected animals. Important human related factors are those related to vaccine design, formulation, and administration. This includes the nature of vaccine, antigen definition and concentration, vaccine stability, expiration date, vaccine material reconstitution, different administration routes (*i.e.*: intranasal, subcutaneous, intramuscular) and schedules of vaccination. Users will also need to be aware of appropriate vaccine storage, shelf life, and the logistics of shipment to different locations. Regarding vaccine-related factors, we should consider first the nature of the antigenic components of the vaccine which may include, the use of infectious pathogens or distinct noninfectious components of the pathogens, purified recombinant antigens representing molecules of the pathogens, or other components, including nucleic acids encoding for protective antigens. Using attenuated forms of infectious pathogens may elicit strong and long-lasting immunity, but this approach may result in undesirable spreading of the infectious agents among animals in the field and may convey the risk for reversion to virulence. Also, the degree of attenuation, either *in vitro* or *in vivo* using live animals, is considered an important feature in the preparation of these types of vaccines. On the other hand, using noninfectious forms, materials, or antigens derived from the pathogens, including killed parasites, purified or partially purified antigen extracts, toxoids, defined recombinant or native subunit vaccines, or nucleic acid-based vaccines, is usually considered safer than the infectious forms, although usually, these approaches are generally less effective compared to live vaccines. Another important factor is the definition of proper, non-toxic, and cost-effective adjuvants, since most non-living vaccines usually require them in order to stimulate adequate and strong immune responses. Finally, deciding on the possible need for vaccine booster doses requires, definition of the characteristics of the vaccine booster, and the number of vaccine inoculations and the frequency required for optimal coverage and efficiency. Importantly, determining these parameters also requires a previous assessment of the quantitative and qualitative responses of the immune systems of vaccinated animals. In addition, the costs associated with the applications of vaccine boosters could be an important consideration to evaluate vaccine practicality.

Achieving, licensing, and marketing efficient vaccines against hemoparasites is also not a trivial endeavor. The relatively slow pace for developing efficient vaccines is due, at least in part, to the typical complex life cycles of the responsible agents, our limited knowledge of protective immune mechanisms, and the fact that parasites are well adapted to their hosts and evolved complex and efficient mechanisms of immune evasion, including antigenic variation. The following sections describe features of selected blood-borne parasites of high impact currently occurring in animals globally (*Babesia*, *Theileria*, *Anaplasma* and *Trypanosoma*), their associated diseases, and different approaches employed for developing vaccines against these pathogens. Also, this review will briefly discuss the current vaccine and control measures of tick and blood-sucking vectors.

There is an urgent need for more research seeking effective strategies to produce durable and effective vaccines for better disease control. Understanding the parasite biology, the host immune responses against the infection, and the smart use of recently developed omics tools will undoubtedly facilitate vaccine development for improved disease control and parasite transmission.

## 2 Brief outlook on selected blood borne pathogens of veterinary relevance

### 2.1 *Babesia* parasites

*Babesia (B)* spp. are apicomplexan parasites that cause Babesiosis and can be transovarial transmitted by different kinds of Ixodes ticks. The disease can affect many mammals, including cattle, horses, sheep, goats, swine, cats, and dogs, and can also be fatal to humans (2). Babesiosis is considered as the second most important blood-borne parasitic disease of animals of veterinary importance, only behind Trypanosomiasis (3). *Babesia* sp. are transovarially transmitted parasites with a complex life cycle involving intracellular stages in at least two types of hosts, vertebrates, and tick vectors. Importantly, *Babesia* parasites undergo sexual reproduction in the gut of their definitive tick host, where they are also able to invade distinct types of cells (such as gut epithelial, ovary, and salivary gland cells), but they are only able to invade the erythrocytes of their vertebrate hosts, which can aid the parasites by providing adequate nutrition and a relatively protected environment for parasite expansion though asexual reproduction. *Babesia* comprises more than 100 species that infect erythrocytes in many vertebrates (4). Babesiosis can affect different livestock in tropical and semitropical regions worldwide, including ovine and caprine, but globally effective and fully safe vaccines against these species remain unavailable. Importantly, *Babesia* parasites have a life cycle that includes sexual reproduction in the midgut of their ixodid tick vectors, which eventually leads to transovarial transmission and the expansion of the parasites in the tick vectors. In this review we will focus mainly on *Babesia* species that infect cattle, equids, and dogs, since they have received more attention for vaccine development.

Bovine babesiosis can be caused by different *Babesia* spp. such as *B. bovis*, *B. bigemina*, *B. divergens*, *B. orientalis*, and *B. major*. In addition, *B. divergens*, which is prevalent in Europe, occasionally can also infect humans (5). However, *B. bovis* is considered as the most virulent *Babesia* spp. responsible for bovine babesiosis (6). This acute and persistent disease poses a significant global challenge for cattle production. Clinical signs range from mild to severe, depending on animal age, impacting animal health and productivity (7), and may include anemia, fever, ataxia, cerebral signs, abortions, anorexia and kidney damage, among others. Young calves ranging from 6–8 months old show some sort of increased resistance likely because they can generate more efficient innate immune responses against the parasites that prevent the devastating effects of acute infections. In contrast, infected adult cattle are more prone to display clinical signs of acute babesiosis, which are presented mainly as fever, anemia, loss of appetite, and weight loss (8). In severe cases of infection by *B. bovis*, animals suffer from neurological manifestations, due to parasite sequestration in the brain blood vessels, which may eventually lead to animal death (7, 8). *Babesia* species can also infect dogs and/or cats, as well as many wild animals, but none of them are known to be of zoonotic importance (9). Canine babesiosis is also a significant tick-borne disease caused by various species of the protozoan genus *Babesia* (10). Large and small forms of *Babesia* species (*B. gibsoni*, *B. canis*, *B. vogeli*, and *B. microti*-like isolates also referred to as “*B. vulpes*,” “Spanish dog isolate,” “*Babesia cf. microti*” and “*Theileria annae*”) can infect dogs with different levels of clinical signs (11, 12). Although canine babesiosis is mostly transmitted by tick vectors, it can also be transmitted to healthy hosts directly by blood transfusion, vertically (13), and by direct contact, as in case of fighting dogs, through wounds, saliva, or blood ingestion (14, 15). Equids also can be infected by *Babesia* spp., *B. caballi*, as one of etiological agents of equine piroplasmosis (EP) which is considered as an economically important disease of equids (16). In addition, EP can be caused by the *Babesia*-related *Theileria equi* parasites, as it will be discussed below, and vaccines remain needed for controlling this disease (16, 17). The small ruminant industry is a very significant component of livestock production especially in developing countries and in poor rural communities worldwide. *B. ovis* is the main causative agent of ovine babesiosis which has major economic impact on small ruminant (sheep and goat) industry in tropical and subtropical areas. Ovine babesiosis is transmitted by *Rhipicephalus* ticks, in particular *R. bursa* (18) and it could result in mortality if animals remain untreated (19, 20).

### 2.2 *Theileria* parasites

*Theileria* (*T.*) spp. are tick-borne apicomplexan parasites responsible for theileriosis, an important disease that affects cattle, equids and other animals worldwide with variable clinical signs. *Theileria* parasites are piroplasmid parasites which are closely related to *Babesia* that can also be transmitted by ixodid ticks. However, there are two key differences among these two parasites: one is the ability of *Theileria* parasites to infect more than a single type of cells in their vertebrate hosts, and another one is their transstadial, rather than transovarial, mode of transmission by ticks. *Theileria* parasites also have complex life cycles, and similar to *Babesia* parasites, they also develop sexual stages, kinetes and sporozoites inside its tick vectors. However, after the infection through the tick bite, *Theileria* parasites develop schizonts and piroplasms once established in their vertebrate hosts (21, 22). In the infected animals, schizonts form merozoites, which in turn parasitize the erythrocytes, which then develop into piroplasms. The piroplasms undergo sexual reproduction after being acquired by ticks during feeding on an infected host. Infected ticks can later infect another host with *Theileria* sporozoite stage parasites by transstadial transmission, regardless of their status of naïve or infected (21). *T. annulata*, *T. parva and T. orientalis* are cattle parasites (23) while *T. equi* and *T. haneyi* infect equines (16). *T. lestoquardi* and *T. ovis* can infect small ruminants (24). *Theileria parva*, *T. annulata*, and *T. orientalis* are the major causes of East Coast fever (ECF), tropical theileriosis, and oriental theileriosis, respectively (23). Based on their ability to infect leukocytes, *Theileria* parasites can be classified as host-cell transforming and non-transforming species. Thus, in the case of *T. annulata*, and *T. parva* infective sporozoites infect leukocytes, to develop into macro-schizonts causing uncontrolled leukocyte proliferation (25, 26). Eventually, these parasites produce merozoites that invade erythrocytes. In contrast, *T. equi* and *T. orientalis* do not induce uncontrolled leukocyte proliferation in horses and cattle, respectively (27). However, *T. orientalis* merozoites invade the host red blood cells, leading to anemia which is associated with the clinical signs of acute oriental theileriosis (28).

### 2.3 Anaplasma

*Anaplasma (A)* spp. is a vector-transmitted rickettsia, that resides in blood cells of its vertebrate hosts, leading to the disease anaplasmosis in tropical and semitropical areas (29). Anaplasmosis can be transmitted among animals by mechanical and/or biological vector transmission. Mechanical transmission may occur at least via two ways, either through contaminated surgical equipment (fomites) carrying the infected blood cells, or by mouthparts of biting flies who carry an *Anaplasma* species (30). Biological vector transmission mainly occurs by the bite of ticks (many different tick species) infected with the blood parasite. Inside the ticks, *Anaplasma* can survive and multiply, or it can stay dormant for months till transmission to another animal through a tick bite (31).

The clinical manifestations of anaplasmosis caused by *A. marginale* and *A. centrale* can be variable and similar to those caused by *Babesia* parasites. Depending on several factors, the intensity of disease can range from mild, lacking clinical signs, to severe, with elevated morbidity and mortality in affected ruminants. However, the infected animals become lifelong carriers that may become reservoirs for the pathogen (32). In addition, *A. ovis* can infect sheep, goats and some wild ruminants causing anaplasmosis (33, 34). Infections by *A. phagocytophilum* (transmitted by *Ixodes ricinus*) in dogs have been described mainly in northern and central Europe while infection with *A. platys* (transmitted by *Rhipicephalus sanguineus*) was identified in dogs from Mediterranean basin Romania, Turkey, Greece, Italy, France, Spain and Portugal (35). Both *A. phagocytophilum* and *A. platys* can infect other animals in addition to dogs, such as cats, sheep, goats, cows, equines, rodents, roe deer, deer, as well as other wild mammals, and even birds, as in case of *A. phagocytophilum* (36, 37). In general, it’s difficult to control anaplasmosis efficiently using vaccination approaches because of the ability of the responsible organisms for undergoing antigenic variation and their genetic variability. Also, the occurrence of multiple hosts and arthropod vectors, as well as the different mechanisms of transmission (biological, mechanical, and transplacental) may also impose important challenges for efficient control (38, 39).

### 2.4 *Trypanosoma* parasites

*Trypanosoma (T)* spp. is also an important blood parasite causing trypanosomiasis in animals and humans. These parasites are kinetoplastids, a monophyletic group of unicellular parasitic flagellate protozoa. Trypanosomiasis is a disease geographically constrained due to the nature of its arthropod vectors with strict requirements in terms of climate and environment. It is known as Surra diseases in South and Central America, Northern Africa, the Middle East, Asia, Indonesia, and Philippines, but it is also known as African animal trypanosomiasis in Central and Southern Africa (40). This disease is considered endemic in at least 37 of 54 countries in Africa (41). It can be transmitted biologically and mechanically by hematophagous insects by biting. The vectors involved in transmission include *Stomoxys*, *Tabanids,* and *Hippoboscids*. However, the parasite can also be sexually transmitted as in equine species infected with *T. equiperdum*. The signs of disease ranged from acute, with high mortalities, to chronic forms, which are frequently concomitant with reduction in body weight, anemia, and infertility. Also, the *T. evansi*’s may cause immunosuppression, usually accompanied with secondary infections, which makes clinical identification more difficult (42). It affects horses, camels, cattle, sheep, goats, pigs and humans. Wild animals also can be infected with *Trypanosoma* parasites, but they rarely suffer from the disease, and can act as infection reservoirs for domestic animals.

In Africa, *T. congolense*, *T. vivax*, and *T. brucei brucei* are the most important trypanosome species affecting domestic livestock in cattle, sheep, and goats, while *T. simiae* can infect pigs, and *T. evansi* infects camels. In South America, *T. vivax* also has an impact on cattle production. While *T. evansi* affects camels in Asia and horses, cattle, and domestic buffalo in South America, India, and Southeast Asia (43). It is interesting to note that *T. evansi* is more common in camels, in contrast to dogs, donkeys, and horses. This may be due to the chronic nature of trypanosomiasis in camels. While infected camels may become weaker and emaciated as the disease progresses, the infection manifests acutely and is usually fatal in dogs and equines (44, 45). Interestingly, although camels as well as donkeys, dogs and horses are similarly exposed to vector challenges, those animals are less prone to be infected by trypanosomes. These may be due to feeding preference of vectors for camels, or the greater ability of those animals to put away the flies through head movements, skin rippling, and other behavioral avoidance mechanisms (46).

In Egypt, *T. brucei evansi* (*T. b. evansi*) is an enzootic organism found in Egyptian camels, which is genetically classified into types A and B (47). *T. evansi* can cause high parasitemia, especially in camels, horses, and dogs (sometimes cattle and buffaloes), and might act both as blood and tissue parasite, because of its ability to invade the nervous system not only in horses and dogs, but also in cattle, buffaloes, and pigs (48, 49). Data on the presence of *T. evans* has been reported in many regions of Upper and Lower Egypt, however, its epidemiological significance in Egypt remains scarce (50).

## 3 Preventive control measures against tick-borne pathogens of veterinary relevance

Vaccines are among the most efficient methods available to prevent infectious diseases. Ideally, vaccines should fully protect the recipient against infections, but this goal is very difficult to achieve against the selected blood borne pathogens discussed hereby. However, current vaccines help ameliorate the risk and impact of acute diseases caused by these groups of selected parasites in animals. Effective stimulation of protective immune responses by vaccines may lead later to a more complete protection in the field, once the animals are exposed to the pathogens. In some specific cases, it could be at least possible to develop vaccines that can prevent infections, for example, by developing vaccines that can block the invading sporozoites, such as is the case of *Babesia* and *Theileria* parasites, preventing the vectors to infest their hosts, or prevent infection of the vectors by the parasites, such as in transmission blocking vaccines. As an alternative (or complementary) to these approaches, there are also research efforts focused on achieving vaccines that can block or control the development of the arthropod vectors, which ideally, could also be combined with pathogen-specific protective antigens.

**Figure 1:**
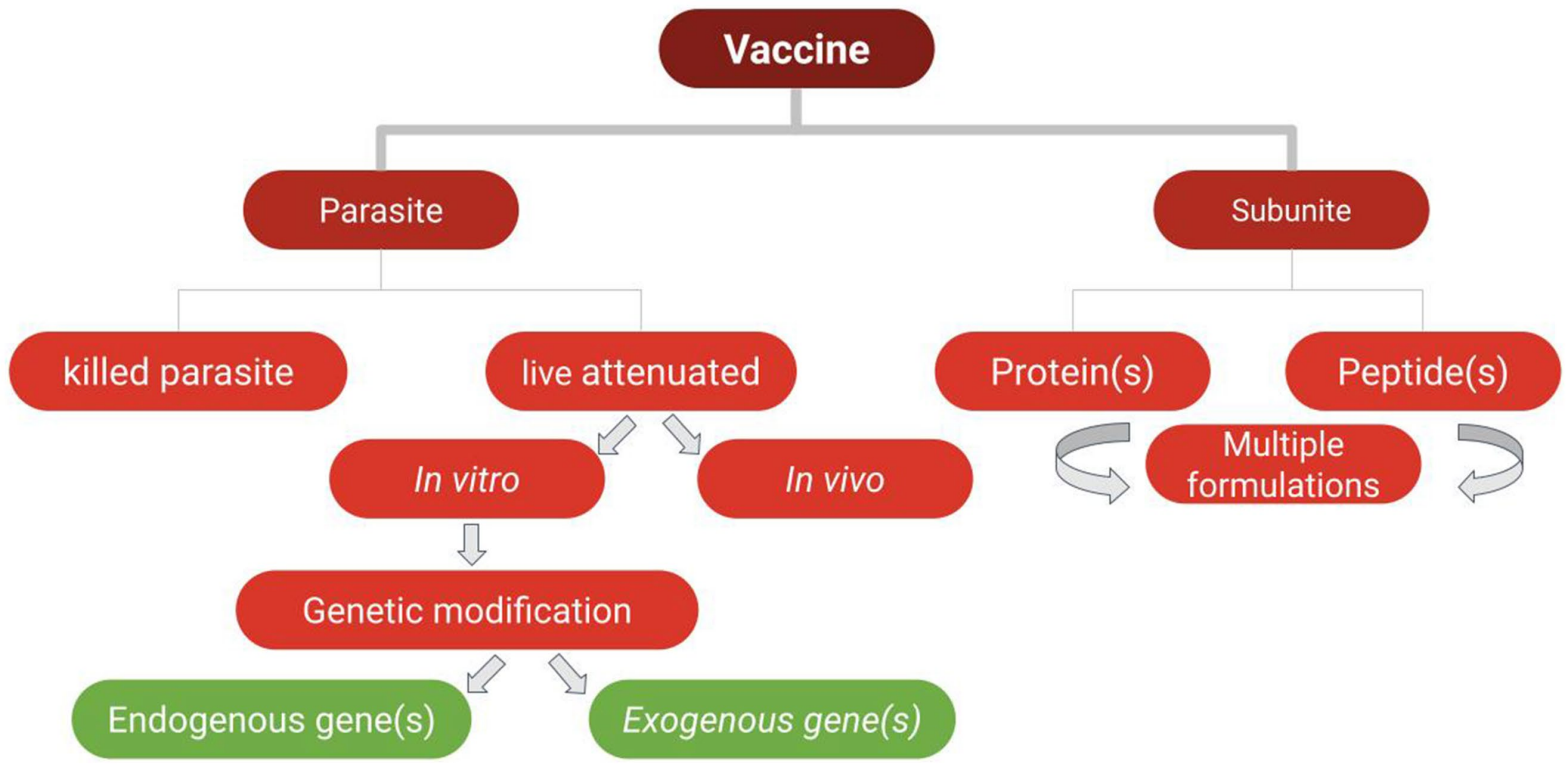
Types of vaccines used in vaccine trials against the selected parasites of veterinary importance addressed in this study.

So far, there are not globally effective, commercially available, vaccines against any of these blood parasites. In the next sections we will address vaccine trials performed against selected parasites of veterinary importance, and some new perspectives in vaccine development against these important blood parasites, mainly inspired by innovations and advancements in cell biology, vaccinology, immunology, and molecular biology, such as gene editing and recombinant protein production. The newly developed experimental vaccines discussed in the sections below typically consist either of subunit vaccines including native or recombinant versions of parasite proteins, crude antigen extracts derived from the parasites, or live parasites that are either attenuated or genetically edited. The goals pursued in the vaccine trials discussed in the next sections vary. While some of these vaccines are aimed to prevent acute disease symptoms, others are designed to prevent parasite transmission (also known as transmission blocking vaccines [“TBV”]). We will also address other approaches aimed at controlling both the parasite and its associated vector, known as dual vaccines (Figure 1).

### 3.1 *Babesia* parasite vaccines

Current measures aimed at the control of bovine babesiosis include individual or combined use of at least three approaches: tick vector control, animal therapeutic treatments, and vaccinations. However, each of these measures has its own pitfalls and limitations. Tick control using acaricidal drugs is a major approach to control babesiosis, but its intensive use invariably leads to the emergence of acaricide-resistant tick strains (51). In addition, this method of control may cause environmental hazards, and the introduction of dangerous chemical residues in the food chain (52). Babesicidal drugs currently used for the treatment of *Babesia* infected cattle, such as imidocarb, may also lead to the surge of drug-resistant parasites, especially when used in suboptimal doses, and worrisomely, the generation of drug residues or metabolites in milk and meat, This features make drug treatments expensive and inefficient as a disease-preventive tool in extensive production systems. Due at least in part, to these limitations, imidocarb is not licensed for use in some European countries (51).

Currently, live vaccines based on attenuated *Babesia* parasites are not available or allowed in many endemic countries. However, some countries with large cattle populations at risk, like South Africa, Argentina, Brazil, Uruguay and Australia opted for producing and using such attenuated vaccines. An important limitation of live attenuated vaccines is their safety, since that they can be potentially virulent, mainly for adult animals, and thus, these vaccines are in use for less than one-year-old calves (53). Moreover, some countries, including Australia and Argentina, use a trivalent vaccine which contain not only attenuated strains of *B. bovis* and *B. bigemina,* but also *A. centrale,* an organism of low virulence originally identified in South Africa (54), that can elicit partial protective responses against *A. marginale*. Trials involving live *Babesia* vaccines are discussed in detail below.

There are also a few vaccine options that are commercially available to prevent canine babesiosis in different countries. The Pirodog® (Merial) vaccine, currently available in the European market is based on soluble parasite antigens obtained from culture media supernatants (55, 56). This vaccine induces partial protection and can reduce severe clinical signs but does not prevent infections. The vaccine is recommended for administration into 5 months of age dogs, with annual booster doses (57, 58). It is worth mentioning that chemoprophylactic approaches are also used as alternatives to vaccination in dogs. For instance, the carbanilide derivative Imidocarb dipropionate has been shown to be effective against *B. canis* infection. The application of a single subcutaneous dose (6 mg/kg) of this drug demonstrated protection for two weeks (59). Doxycycline (5 mg/kg/day) also reduces the severity of disease in dogs that experimentally infected with an extremely pathogenic isolate of *B. canis* (60).

The following sections describe recent efforts toward developing vaccines against *Babesia* parasites using different approaches, including subunit, whole parasite, and culture supernatant vaccines (Figure 1).

#### 3.1.1 Vaccine trials against *Babesia* based on subunit and native crude extracts antigens

Researchers have identified several potential recombinant and native subunit and crude antigenic extracts that have been proposed as vaccine candidates for bovine babesiosis, since they can prevent severe clinical signs (Table 1). Crude extract antigen and subunit vaccines have been designed and tested in several reported *in vivo* trials.

**Table 1:** Different forms of subunit and native crude extract vaccine trials against blood stage of *Babesia* parasites.

Vaccine form	Efficacy	Reference
Native protein		
*B. bovis* native extract: Combination of two affinity chromatography purified antigens (11C5 and 12D3)	Immunization of cattle resulted in decreased parasitaemia upon challenge with homologous *but* not with heterologous virulent *B. bovis* strain indicating these antigens induce strain-specific immunity.	(148, 149)
Adult sheep vaccinated twice with crude extracts of either *B. bovis* or *B ovis* parasites (*In vitro* culture extract)	Both vaccinated groups had significantly reduced parasitaemia upon challenge with *B. ovis* organisms.	(150)
Exoantigen-containing supernatant fluids of *in vitro B. bovis* and *B. bigemina* cultures	Vaccination reduced the incidence of clinical disease among vaccinated animals and complete protection against mortality caused by babesiosis.	(151)
Recombinant protein		
*B. ovis* apical membrane antigen-1 (rBoAMA-1)	Experimentally vaccinated sheep generated a specific response against the recombinant protein, but the antibody response did not associate with protection upon challenge with *B. ovis* infected cell culture.	(20)
Recombinant Rhoptry associated protein 1 (RAP-1) of *B. bovis.*	Vaccination did not elicit protective immunity against challenge with the virulent *B. bovis* strain.	(152–156)
Recombinant Merozoite surface antigen-1 (MSA-1) of *B. bovis.*	Vaccination did not protect calves after challenge with the *B. bovis* T2Bo strain	(157)
Apical membrane antigen 1 domain [rBbAMA-1(I/II)]	Antigens induced strong Th1 cell responses. No challenge with virulent *B. bovis* reported.	(158, 159)
Mixture of MSA-1, MSA-2c and 12D3 recombinant proteins emulsified with the Montanide adjuvant, administered in two doses.	No signs of effective protection after challenge with the *B. bovis* Yucatan strain	(160)
*B. bovis* RON2 containing conserved B-cell epitopes	Vaccine stimulated antibody production in cattle which inhibited *in vitro* culture parasite invasion. No challenge with live pathogenic *B. bovis* reported.	(161)
Peptide form		
Traditional form		
*B. bovis* AMA-1, MSA-2c and RAP-1 containing conserved B and T-cell epitopes	Production of neutralizing antibodies in cattle and durable Th1 immune response. No challenge with live pathogenic *B. bovis* reported.	(162)
*B. bovis* merozoite surface antigens: MSA-2a1, MSA-2b, and MSA-2c	The vaccine elicited immune stimulation in vaccinated cattle. No challenge with live pathogenic *B. bovis* reported.	(163)
Chimeric multigene vaccine (viral vector)		
Chimeric multi-antigen of DNA fragments containing B- and T-cell epitopes of merozoite surface antigen 2c (MSA-2c), rhoptry-associated protein 1 (RAP-1) and heat shock protein 20 (HSP20) genes.	All vaccinated cattle showed clinical signs of disease upon challenge with virulent *B. bovis* strain	(164)
Poly-N-acetylglucosamine (PNAG)		
Synthetic ß-(1 → 6)-linked glucosamine oligosaccharides conjugated to tetanus toxoid (5GlcNH2-TT)	The vaccinated calves were not protected upon challenge with virulent *B. bovis* parasites.	(62)

Typically, a subunit vaccine contains selected purified molecules derived from a target pathogen that are antigenic and able to elicit a protective immune response. These antigens may be either purified native or recombinant proteins, or in other forms, as described below. Unlike live attenuated or non-viable parasite derived vaccines (such as irradiated, or chemically inactivated), subunit vaccines typically include only specific and selected components such as proteins, polysaccharides, or peptides (61). As such, subunit vaccines are considered safer and more stable than live vaccines because they do not contain infectious components.

Because this property applies to these two types of antigens, vaccine trials involving subunit and native crude extracts derived from the parasites, are included together in this section.

Subunit vaccine candidates can take various forms, including purified recombinant protein(s), and/or synthetic peptide(s) representing relevant B and T cell epitopes, or mixed formulations. Also, custom designed synthetic genes encoding for a combination of several protective B and/or B and T cell epitopes can also be used for expression in prokaryotic or eukaryotic systems (62), or even for direct injection of coding DNA into the animals using specific devices such as gene guns (Table 1). In addition, animal vaccine trials have also been performed for experimental subunit vaccines based on recombinant proteins aimed at blocking pathogen transmission (Table 2).

**Table 2:** Different forms of subunit vaccine trials against sexual stage of *Babesia* parasite (Transmission blocking vaccine).

Antigen(s)	Efficacy	Reference
Mixed recombinant proteins		
*B. bovis* 6cys A and B	Failed to block transmission *in vivo*	(52)
Single protein		
*B. bovis* HAP2 (hapless2)	Vaccination blocked *Babesia* transmission via tick in *in vivo* experiments performed on immunized cattle.	(147)
*B. bigemina* HAP2	Anti-HAP2 specific antibodies from immunized animals were able to block zygote formation *in vitro* for *B. bigemina.* No *in vivo* application followed by a challenge described.	(165)
Peptides		
*B. bovis* 6cys A and B	Rabbit anti *B. bovis* 6cys A and B were able to block *B. bovis* gametes development *in vitro*.	(52)

Several strategies for selecting protective blood stage or transmission-blocking antigens were proposed, with some focused on functionally relevant molecules and others using pragmatic approaches like previously defined surface exposed, neutralization sensitive and conserved molecules. Protection in blood stage vaccines is typically defined by the ability of vaccinated animals to survive standardized challenges from *Babesia* merozoites from virulent strains, and the results and efficiency in these trials are usually compared to protection resulting from live vaccinations. The following Tables 1, 2 illustrate different vaccination trials for blood and sexual stage subunit and crude antigenic extracts so far described in the literature.

Regarding blood stage vaccines, none of the subunit vaccination trials targeting blood stage parasites described in the literature were able to elicit fully efficient and long-lasting protection against challenge with virulent *Babesia* strains. Some of these unsuccessful results may be due, at least in part, to the structural and antigenic differences between native and recombinant versions of the antigens, including parasite-specific protein folding and post-translational modifications of the native proteins, which may result in critical differences in their antigenicity, when compared to recombinant proteins produced in heterologous expression systems (63). For instance, immunization experiments using native purified *B. bigemina* antigens, including RAP-1 of *B. bigemina* resulted in partial protection (64).

There are other issues that might significantly influence the outcome of the blood stage vaccine trials involving recombinant proteins and synthetic peptides described in Table 1. One important factor is the nature and severity of the challenge used. While in nature animals get usually infected by ticks, which inoculate variable amounts of antigenically distinct sporozoite stage parasites in the blood of the vertebrate host, the challenges in all these trials were invariably performed using a large, fixed, amounts of blood stage merozoites from a defined highly virulent strain of *Babesia*. The current method of challenge using large numbers of virulent merozoites may be realistic for assessing immunity elicited by live vaccines, but that may not be the case for other cases involving recombinant proteins. We thus suggest that a more realistic and standardized method of parasite challenge, preferably using sporozoite stage parasites or natural *Babesia*-infected tick field challenge, should be also developed and adopted for future trials.

However, in case of TBV, while the full-size *E. coli*-derived recombinant version of the *B. bovis* Hap2 protein was able to block transmission via ticks, that was not the case for full size eukaryotic system-derived r6cys proteins. It was hypothesized that the transmission-functional regions of these proteins were poorly immunogenic when presented in the context of the full- size 6cys A and B proteins. This notion was supported by additional experiments that identified specific and poorly immunogenic transmission-sensitive regions of these proteins that may contain epitopes responsible for transmission reduction function (52). Future vaccination experiments using these specific regions might result in the definition of alternative candidates for TBVs based on 6cys proteins.

Worthy of note that there are other promising functional approaches toward *Babesia* vaccine candidate definition that had been proposed in the last few years (65). One approach is based on the selection of critical protein-derived conserved regions related to parasite adhesion. Interestingly, conserved high activity binding peptides (cHABPs) to the erythrocytes were identified in the genome of *B. bovis* using intensive *in silico* analysis (66). Similarly, using a bioinformatics approach researchers identified a sub-immunodominant B-cell epitope in a highly conserved 15 amino acid region of the Rhoptry Associated Protein Related Antigen (RRA) of *B. bovis*, that is also a candidate for inclusion in peptide-based subunit vaccines (67). These *in silico* subunit vaccine antigen candidate identification approaches, could also be extended not only against *Babesia* but also against other blood parasites, due to the availability of annotated genomes for most parasites of medical importance, with the addition of other omics analysis. Moreover, exploiting exosomes, which consists of small extracellular vesicles that serve as carriers for proteins, nucleic acids, and lipids in host-*Babesia* or parasite–parasite interaction should be also considered (68, 69). This approach was successfully applied in *Plasmodium* spp., and it allowed the identification of novel subunit vaccine candidates involved in non-classical protein secretion, pathogenesis, immune modulation, and parasite–host interactions (70). Its potential in disease pathogenesis was explored in two major human *Babesia* species, *Babesia divergens*, from *in vitro* culture and those from an *in vivo B. microti* mouse infection (71). Thus, further similar studies using this approach in *Babesia* vaccination are warranted.

#### 3.1.2 Vaccine trials based on whole *Babesia* parasites

Using whole *Babesia* live or inactivated parasites in vaccination approaches has important advantages, since they should include most, if not all, antigens expressed at least in certain stages of the organisms, and in some cases, such as in live attenuated vaccines, they may overcome limitations due to antigenic variation and natural polymorphism (72), although this may depend on the method used to reduce the virulence of the parasites. These types of vaccines may contain live attenuated or genetically modified parasites, or whole inactivated (killed) parasites.

##### 3.1.2.1 Inactivated (killed) parasite

Killed vaccines were achieved in different ways. One of those is the freeze-dried suspension of *Babesia argentina* (*B. argentina*) (currently known as *B. bovis*) parasite (73) and *B. microti*-killed parasites enclosed within liposomes which incorporated a mannosylated lipid core peptide. In both cases the vaccination induced protective immunity in cattle and in mice against *B. argentina* or *B. microti, respectively.* Also, chemical attenuation was performed on *B. microti* parasitized red blood cells from infected mice by using Tafuramycin-A (TF-A). A culture-based liposomal vaccine, a liposome containing killed parasite material, was also used as a vaccine in mice. The parasitemia was reduced in vaccinated animals upon challenge with the *B. microti* (74).

##### 3.1.2.2 Live attenuated vaccines

Live vaccines based on attenuated parasites remain as the only preventive measure against bovine babesiosis caused by *B. bovis* and *B. bigemina,* and for canine babesiosis caused by *B. gibsoni*.

These live vaccines have several significant limitations, including the need of cold chain for transportation and short shelf-life, which is only about of 4 days from the production date when stored between 2 and 8°C. Also, live attenuated parasites strains may pose the risk of reversion to virulence upon exposure to the natural conditions in the field (52). Moreover, there is also the risk of transmitting other contaminating blood-borne pathogens during vaccination. This may occur because the process of attenuation requires serial infections passages on several splenectomized or spleen intact calves (for *B. bovis* and *B. bigemina* respectively) (75), and the large-scale production of the vaccine in some countries (*i.e.*: Australia) involves infecting splenectomized cattle as well. Yet, the method of vaccine production varies among countries, and in some cases such as in Argentina, the attenuated strains are expanded in *in vitro* cultures, rather than in splenectomized cattle, for vaccine production purposes. Finally, as mentioned before, there is an age restriction for the vaccination, and issues regarding dose standardizations (54).

Attenuation can be achieved from virulent *B. bovis, B. bigemina,* and *B. gibsoni* parasites by using different approaches (Tables 3, 4). Attenuation can be generated *in vivo* through successive rapid passages in splenectomised calves, in the case of *B. bovis* (76), or by slow passages in non-splenectomized calves for *B. bigemina* (77).

**Table 3:** *In vivo* live attenuated parasite used in vaccine against *Babesia*.

Vaccine component	Efficacy	Reference
South African S24 vaccine attenuated strain (with 24 passages in cattle) of *B. bovis*	Vaccination conferred protection in cattle and the attenuated strain was not transmissible by ticks. Co-transmission of the attenuated strain together with field strains resulted in the emergence of a new transmissible parasite population with a distinct hybrid genotype.	(166, 167)
Australian *B. bovis* vaccine attenuated strains (Ka strain), was produced by rapid 20–30 passages in cattle.	This strain is used as a component of a trivalent live vaccine which also contains an attenuated *B. bigemina* Australian strain, and *Anaplasma centrale* which adds protection against *Anaplasma marginale*.	(54)
Test the infectivity of a vaccinal and a pathogenic strain of *Babesia bovis* for the tick *Boophilus microplus*. Vaccine strainR1A isolated from clinical case and attenuated and produced by 30 passages in splenectomised cattle. Pathogenic strain of *B. bovis* S2P isolated from splenectomised calf infected naturally via tick larvae by *B. bovis*.	Engorged female ticks fed on calves inoculated with the S2P strain were able to transmit the infection to splenectomised calves. This vaccine strain was shown not to be transmissible by ticks under natural conditions.	(168)
Attenuated *B. bigemina* (*in vivo* and *in vitro*) were used to vaccinate two groups of Holstein Friesian heifers. Another group of heifers was vaccinated twice with purified soluble antigens obtained from the supernatant of *in vitro* culture combined with saponin.	Heifers vaccinated with attenuated *B. bigemina* either *in vivo* or *in vitro* resisted challenge without specific treatment, whereas the opposite occurred in heifers group vaccinated with culture soluble antigens. Antibody titers were higher in heifers inoculated with soluble antigens than in heifers inoculated with *in vivo* live *B. bigemina*, suggesting that antibody titers may not be a proper indicator of animal protection.	(169)
An australian *B. bigemina* vaccine strain (G strain) of reduced virulence was tested in animal against a virulent South African strain	The strain caused mild reactions in 10 animals and afforded good protection to challenge with a virulent strain.	(170)
An Australian vaccine strains (including *B. bovis*, *B. bigemian* and *A. marginale*) was test their safety in pregnant Holando heifers and their efficacy against challenge from inoculated local field strains of the three parasites from Paraguay	The *Babesia* strains, but not *A. marginale*, provided good protection against field challenge and were safe to be used in highly susceptible cattle.	(171)

**Table 4:** *In vitro* live attenuated parasite lines used in vaccines against *Babesia*.

Vaccine component	Efficacy	Reference
Infected erythrocytes of *B. ovis*/Akçaova stabilate (passage 5 in *in vitro* culture) was used for sheep immunization, either alone or mixed with *B. ovis* rBoAMA*-*1 protein.	Sheep co-immunized with sublethal dose of *B. ovis*/Akçaova stabilate and *B. ovis* rBoAMA*-*1 protein did not show clinical signs and/or changes in hematological parameters following challenge with *B. ovis* parasites.	(20)
A *B. bovis* isolate was cloned by *in vitro* cultivation and compared to the original cultured isolate for pathogenicity by yearling heifers inoculation. Four of them were inoculated with cloned material and the other 4 with the original culture.	The four animals receiving the cloned parasite showed comparatively minor hematologic changes and no clinical signs. One animal died in the group vaccinated with the original culture. All the animals receiving the cloned parasites were totally immune with no significant change in temperature or decrease in PCV upon challenge 100 days after vaccination.	(172)
Attenuated *B. bovis* strain by *in vitro* culturing using equine and bovine serum	All four splenectomized vaccinated calves recovered from mild clinical disease signs, and developed solid immunity upon challenge with virulent strain.	(173)
Possible cross-protection conferred by strains of *B. bigemina* or *B. bovis* derived from *in vitro* culture was tested in cattle by vaccination of calves using individual (*B. bovis or B. bigemina*) or combined (*B. bovis* and *B. bigemina*) live parasites.	The resulted protection was 25, 50, and 100% for cattle immunized with *B. bigemina, B. bovis*, and *B. bigemina/B. bovis*, respectively upon natural challenge via *Boophilus microplus* tick in the field. The mixed vaccinated animals were challenged under control conditions with virulent strains of both protozoan species. All vaccinated animals survived and showed a slight decrease in PCV with unchanged rectal temperature.	(174)
*In vitro* culture derived *Babesia bovis* and *B. bigemina* vaccine to susceptible *Bos taurus* bulls in a babesiosis endemic area was evaluated. Animals were over 18 months of age	After vaccination for 21 days, all animals under the study were released to a tick infested paddock where they remained until the end of the study. Results showed that a combined *B. bovis* and *B. bigemina* vaccine can confer a 70% protection to bovines introduced to *Babesia* infected areas.	(175)
Co-immunization of cattle with a vaccine against babesiosis (*B. bovis* and *B. bigemina*) and *Lactobacillus casei*	Results suggested that the vaccine efficiency was in part improved due to the *L. casei* boost of IgG1 over IgG2 antibodies agaisnt *B. bovis* and *B. bigemina*.	(176)
Vaccine with *Babesia bovis* and *B. bigemina* cultured *in vitro* maintained in a bovine. Serum and serum-free medium	The vaccine derived from *in vitro* culture in bovine serum-free medium reached 100% protection *vs* 83.3% protection with a vaccine derived from *in vitro* culture in bovine serum medium.	(177)
Long-term (LTCP) cultured *B. bovis* parasites [Attenuated S74-T3Bo-12 years]	Cattle immunized by this strain showed mild clinical signs, and the parasite wasn’t transmissible in a tick transmission experiment (TBV).	(6)
	The immunized 6 months old calves survived superinfection with Vir-S74-T3Bo without displaying signs of acute babesiosis.	(7)
	In a separate experiment, immunized adult (>1.5 year of age) cattle displayed self-limiting signs of acute infection and protected against challenge with the homologous virulent *B. bovis* strain Vir-S74-T3Bo.	(8)
Culture-derived attenuated live vaccine against *B. bigemina*	A single shot of attenuated vaccine was capable of complete protection and parasitic clearance after the challenge with a lethal intravenous challenge virulent calf-derived *B. bigemina*	(178)
*B. gibsoni* attenuated Oita isolate 400 *in vitro* culture passages	Vaccinated dogs with the attenuated strain were protected against the challenge with *B. gibsoni* virulent Oita isolate	(179)

Attenuation can also be achieved in *B. bovis* by long term *in vitro* culturing of virulent parasites (6), by deep freezing for long periods in liquid nitrogen (78), using chemical treatments, or by irradiation (79). Tables 3, 4 show previously published vaccination trials performed using *in vivo* and *in vitro* live attenuated parasites in vaccine trials.

In general, live vaccines against cattle babesiosis are not recommended for use in adult animals, since they may induce acute disease. However, the three experimental *in vitro* culture immunizations (6–8) performed in cattle described in Table 4 suggest that the attenuated S74-T3Bo is also a potentially efficient and sustainable attenuated candidate vaccine strain not only because was able to prevent acute bovine babesiosis upon challenge with a homologous virulent strain in highly susceptible adult cattle and young animals, but also because it was shown to be non-tick transmissible. However, the effectiveness of this vaccine still needs to be tested against heterologous virulent strains. In summary, this *in vitro* cultured attenuated strain might become an optimal choice as a component of attenuated live vaccine because it is a clonal-like strain (6), and thus might be unlikely to revert to virulence. Nonetheless, more experimental vaccinations tests using the LTCP attenuated S74-T3Bo strain are still needed in a larger number of animals and using different challenge strains. Similarly, *in vitro* culture attenuated strains of *B. bigemina* and *B. gibsoni* (Table 4), may be also optimal candidates for “universal” and more standardized effective *in vitro* culture attenuated vaccines, but again, more testing, including studies on tick transmissibility, are warranted on these strains.

##### 3.1.2.3 Genetically modified *Babesia* parasites

The advancements in the field of the gene editing, including transfection technologies and CRISPR/cas9, in combination with *in vitro* culture systems, and other related technologies, may lead eventually to the generation of genetically modified live vaccines. This type of vaccines can serve as dual vaccines, targeting both the parasites and their vectors, in an integral approach of control. Still, there are important challenges for approving, producing, and commercializing this type of vaccines. In addition, there is the need for large scale cultivation to produce the parasite, and being a live vaccine, its distribution may also generate costly and cumbersome logistic requirements, such as the need for a cold chain (80). Another limitation of this approach includes concerns derived from the application of the genetic modified vaccine into the field animals. Despite these potential obstacles, this approach may prove to be cost-effective under certain circumstances. The following scientific trials shown in Table 5 exemplify the innovations achieved in this area of research.

**Table 5:** Gene modification studies in vaccine development against *Babesia* parasite.

Type of vaccine	Study	Target	Reference
Stable transfected strain of *B. bovis* expressing an enhanced GFP (eGFP) and a chimeric version of Bm86 (*B. bovis*/Bm86/eGFP)	Splenectomized calves immunized with *B. bovis*/Bm86/eGFP showed mild signs of acute disease after challenge with *B. bovis* and generated antibodies that recognized native Bm86, a vaccine candidate protein expressed in the vector tick midgut. The number of ticks that fully developed and detached as engorged females was reduced (70%) in vaccinated calves. PCR analysis for *B. bovis* in ovaries and eggs of female ticks fed on immunized calves was negative.	*B. bovis* blood stage. *R. microplus & R. annulatus*	(180)
Live *B. bovis* vaccine expressing the protective tick antigen glutathione-S-transferase from *Haemaphysalis longicornis* (HIGST)	Cattle inoculated with transfected parasites developed mild babesiosis upon challenge with a virulent strain of *B. bovis* and produced antibody responses against the tick antigen expressed by the transfected parasites. Challenge of vaccinated cattle with heterologous *R. microplus* ticks, resulted in reduction of egg fertility and weight of fully engorged female ticks. Reduction in tick size and fecundity of *R. microplus* was also observed.	*B. bovis* blood stage and *R. microplus* ticks.	(181)

Both trials described in Table 5 presented evidence for the ability of transfected live attenuated parasites to protect against challenge with a virulent parasite strain. At the same time, the parasites used in these vaccination trials will not be able to be transmitted by competent ticks because these experimental vaccines are based on long term *in vitro* cultured *B. bovis* that likely lost their transmission phenotype (6). Moreover, the addition of a gene expressing protective antigens derived from the tick vectors to the *B. bovis* vaccine strain, increases the advantages of this live-vaccine approach by providing a dual effect against the pathogen and its vector.

### 3.2 *Theileria* parasite vaccines

The main strategies currently used to control theileriosis are based on the use of anti-*theilerial* drugs. Also, in the case of bovines, improved control includes the use of indigenous and cross breeds of cattle that are known to be more resistant to the parasites and its vectors, such as *Bos indicus* (21). Historically, vaccines based on attenuated macro-schizont cell lines were successfully used in case of *T. annulata*. However, since these are attenuated parasite lines, there is a concern of spreading infections in the field (81). Table 6 shows different antigens used in subunit, or combined subunit-live vaccine trials against *Theileria* parasites.

**Table 6:** Vaccine trials based on *Theileria* antigens associated with different parasite developmental stages.

Form	Efficacy	Reference
*T. annulata*		
Recombinant SPAG-1 [Surface sporozoite antigen] & TAMSA-1 [Merozoite surface antigen]	Vaccination resulted in significant levels of protection in cattle when the two antigens (SPAG-1& TAMSA-1) were used in the form of a cocktail. The immunization results indicated a potential synergistic effect between both antigens in inducing protection against *T. annulata.* Also, in contrast to using SPAG-1 alone, immunization with TAMS-1 reduced the severity of several disease parameters compared to non-immunized control animals.	(182, 183)
Combination of SPAG-1 and the attenuated parasite cell line	All immunized animals survived challenge with virulent parasites. Protection provided by combining sporozoite and schizont antigens in vaccination against the disease was considered as effective.	(184)
Ta5 and Ta9	These antigens proved that the role of cytotoxic cells is less evident in the protection to *T. annulata* than to *T. parva*. There was no parasite challenge reported.	(185)
6-cys antigen	Candidates for transmission blocking vaccine were identified by bioinformatic analysis, but without animal trial experiments.	(186)
*T. parva*		
Infection and treatment method (ITM)	ITM is considered a primary choice to prevent the dramatic effects of East Coast Fever. The procedure involved inoculation with a cocktail of *T. parva* strains followed by administration of long acting oxytetracycline. This method induces protective immune responses.	(81, 187)
Polymorphic immunodominant molecule (PIM). An expressed protein by both the sporozoite and schizont stages of *T. parva.* Used with different delivery platforms *i.e.* nanoparticles	This vaccine elicits strong humoral and cellular immune responses in vaccinated animals, but no protection was reported.	(188, 189)
*T. parva* p67 (A surface sporozoites antigen)	Immunized animals showed variable levels of protection in field trials following challenges with parasite sporozoites.	(190, 191)
Polypeptide derived from p67 C-terminal by using nanotechnologies	Although vaccination showed variation in the response among the immunized animals, it displayed significant protection	(192)
Tp1	This antigen induced schizont-specific CD8 (+) central memory T cells with partial protection against a lethal challenge (36% survival)	(193)
*T. orientalis*		
Live vaccine using piroplasm parasites	Has low efficacy and represent a risk for parasite transmission	(23)
Full-length or immunogenic segments of the *T. orientalis* major piroplasm surface protein (MPSP). This protein is highly diverse and involved in immune evasion.	The immunized animals show no clinical signs with lower parasitemia level when challenged with sporozoites	(194)
*T. equi*		
Passively transferred merozoite-specific IgG3 [immune plasma containing *T. equi* merozoite-specific antibodies infused into young horses (SCID foals)]	Although the immunized animals were infected after intravenous challenge with homologous *T. equi* merozoite stabilate, foals show a delayed time to peak of parasitemia and significant delay in the clinical signs.	(195)
*T. equi* recombinant EMA-2 [geldings- pregnant mares and foals]	The vaccinated animals showed humoral and cellular immunity responses similar to those observed in natural parasite infections. Vaccinated animals survived challenges with *T. equi*.	(196)

Because of the current lack of commercial vaccines to control animal theileriosis, the variable therapeutics effectiveness (82), and the results derived from unsuccessful previous vaccination attempts, the main current control strategies against *T. parva*, *T. annulata*, and *T. orientalis,* in cattle, and *T. equi* in equines, still rely mainly on the control of their tick vectors. Ideally tick control should be done using environmentally friendly acaricidal drugs or tick vaccines, which can be complemented by parasite control using anti-*theileria* drugs. However, none of these options are currently available and more research in these fields is required.

Although the current use of ITM and cell line vaccines to control ECF and tropical theileriosis, respectively, are relatively effective, the procedure still has drawbacks. This includes the need for a cold chain for distribution, high costs, and the potential risk of tick transmission of *Theileria* parasites (83). Since ITM depends mainly on the use of oxytetracycline, there is also the concern of developing and expanding antibiotic resistances through food and milk contamination (84). The most widely used vaccines against *T. annulata are attenuated* schizont cell culture. The methodology of production and its safety evaluation had been evaluated (85, 86). This kind of vaccine is used in different countries such as Israel, Iran, Turkey, Spain, India, northern Africa, central Asia and the People’s Republic of China (87). A recent study in Egypt reported a vaccination trial in cattle using culture-attenuated schizont-infected cell lines isolated from Egypt. The vaccinated groups were inoculated with 4 mL (1 × 10^6^ cells/ml) of the attenuated cell line. Three weeks after vaccination, calves of vaccinated and control non vaccinated groups were transported to the New Valley Governorate (Egyptian oasis), where they were kept under field conditions and exposed to natural *Theileria annulata* challenge. All animals in the control unvaccinated group showed severe clinical signs and died despite treatment with buparvaquone. In contrast, animals in the vaccinated group became seropositive without developing severe clinical signs other than transient fever. These findings indicate that the Egyptian attenuated cell line was successful in protecting against tropical theileriosis under field conditions (88). Although, cell culture vaccine against *T. annulata* has been recognized for more than three decades and has proven to be effective in the field, it still has limitations (87), and each country developed the vaccine from local isolates (89). Because of the continuous attenuation, some of the attenuated lines lost the ability to differentiate to erythrocytic merozoites (piroplasms) when inoculated to cattle, one example of that when *Hyalomma* nymphs fed on vaccinated cattle did not become infected (90). This pitfall, in addition to the problem in standardization of the antigenic composition of the cultured parasites and the need of a cold chain for distribution of the vaccine to the field are limiting factors in commercialization of this vaccine (89).

*T. equi* and *B. caballi* are both responsible for equine piroplasmosis, a disease that limits horse movement worldwide and seriously affects the development of horse industries and equestrian sports. Equine theileriosis caused by *T. equi* is mainly prevented in non-endemic areas worldwide by regulating the movement of horses from endemic countries. Also, for some countries, animals must be tested negative for *T. equi* serologically, as an obligatory procedure in animals’ importation (91). Thus, in order to generate potent vaccines, research should be focused ideally on targeting different parasite developmental stages, such as blood and sexual stages, and avoiding, if possible, antigens that may interfere with the current mandatory tests required for exportation of horses. Also, advances in reverse vaccinology should be applied. The similarities among different species of *Theileria* parasites genomes should be considered during vaccine approach. Importantly, identification of host immune correlates which are associated with protection against the parasite infection, would be of high value for vaccine design. Using effective adjuvants and innovative and practical vaccine delivery systems should also be considered as crucial aspects of vaccine development.

### 3.3 *Anaplasma* vaccines

Although *Babesia* parasites are apicomplexan protozoa and *Anaplasma marginale* is a rickettsia, they were historically linked since they form part of a complex of diseases also known as cattle tick fever or Bovine parasitic sadness (BPS) in several countries, sharing important features: they both infect exclusively erythrocytes in the vertebrate hosts causing a clinically related acute disease, and they are all transmitted by *Rhiphicephallus* (*Boophilus*) ticks, often co-occurring in endemic areas.

There are currently no worldwide effective vaccines to prevent canine anaplasmosis, caused by *Anaplasma phagocytophilum* and *A. platys,* nor for bovine anaplasmosis caused by *A. marginale* (92, 93). *A. phagocytophilum* has zoonotic potential and is responsible for human granulocytic anaplasmosis.

Many vaccine candidates have been evaluated for the control of bovine anaplasmosis, but the antigenic diversity of the pathogen has impaired many efforts to control the disease (92). However, a live heterologous vaccine based on *A. centrale* have been used since the early 20th century. *A. centrale,* is an organism isolated in South Africa, which is less virulent for bovines than *A. marginale,* and provides some degree of cross-protection against this pathogen in naive cattle. In some countries, *A. centrale* is added to live *Babesia* spp. vaccines, to make a trivalent vaccine (including *B. bovis, B. bigemina*, and *A. centrale*). This trivalent vaccine is in use as a live vaccine in countries such as South Africa, Australia, Argentina, Brazil, Uruguay (94). Despite the success of this vaccine to some extent, there are few reports indicating *A. centrale* was the cause of vaccine outbreaks, with fatalities (95). Furthermore, in some cases the vaccine failed to induce immunity against *A. marginale* challenge (96). Moreover, University Products LLC (Louisiana, USA) currently offers the only inactivated commercial vaccine against bovine anaplasmosis based on *A. marginale*^1^ (97). This vaccine has been field-tested for over 20 years (98). Although this vaccine does not prevent infection with virulent *A. marginale,* it induces enough protection in cattle against acute anaplasmosis (92). This vaccine requires only two doses in the first year, followed by one annual booster each year, and it is safe to be used in any stage of bovine pregnancy (98). Still this inactivated vaccine is not approved in many countries, but it is approved and established for use in the United States. The following sections provide a glimpse of some vaccine development efforts in the field of *Anaplasma* (Figure 1).

#### 3.3.1 Whole organism vaccines

In this section, *A. marginale* vaccines based on whole organisms were introduced, which can be inactivated or live vaccines. Inactivated vaccine trials are shown in Table 7. The live vaccine contained the virulent form of the pathogen in a low dose, which may be derived from infected animals, or attenuated through multiple animal passages, or in long term *in vitro* culture. In case of *Anaplasma,* the mammalian *in vitro* culture system efforts, based on erythrocytes, have been explored without any success, either using bovine erythrocytes alone, or co-cultured with endothelial cells [bovine pulmonary artery] (99). Only several tick cell lines were successful to establish the *Anaplasma in vitro* culture for both *A. marginale and A. centrale*, but long-term *in vitro* cultures of these organisms in erythrocytes was not yet achieved (100, 101). However, cattle immunization with *in vitro* cultured *A. marginale* induced an antibody immune response but without the expected protection level (102). The trials of live *Anaplasma* vaccines are shown in Table 8.

**Table 7:** Inactivated vaccines against cattle anaplasmosis.

Form	Efficacy	Reference
Lyophilized highly infected RBCs in oil adjuvant and inoculated into susceptible hosts	Not a practical formulation due to high content of erythrocyte stroma.	(94, 197)
Inactivated preparations of purified initial bodies from bovine RBCs	The vaccine was not effective in immunized animals against a heterologous strain.	(198)
Vaccination with three strains that shared major surface proteins Msp1a and Msp4	Vaccinated animals that received the challenge with the homologous strain in the form of inoculum showed no protection and chemotherapy was required to prevent death. In contrast, animals that received the challenge by infected ticks (gradual infection), were protected against naturally tick-transmitted anaplasmosis.	(199, 200)

**Table 8:** Live vaccines against cattle anaplasmosis.

Form	Efficacy	Reference
live virulent *A. marginale* paired with treatment premunition	Variable. Some vaccinated animals were infected with a delayed incubation period, while other animals showed no clinical signs after vaccination.	(201)
Virulent strains of *A. marginale* attenuated by passaging organisms in unnatural hosts (splenectomized sheep and deer)	Variable. The deer-passaged strain vaccine failed to induce solid protection in vaccinated animals while sheep-attenuated strains induced protection.	(201, 202)
Fresh or frozen infected RBCs with *A. marginale* strains of naturally low virulence	Induced a mild clinical syndrome in the immunized animals	(203, 204)

#### 3.3.2 Subunit vaccines against *Anaplasma* organisms

Novel omics technology has yielded a list of antigens that could help the researchers to explore new possible vaccine candidates. Protein function, localization, conservation, and either their dominant or subdominant antigenic characteristics, are the main criteria, among others, for antigen selection. Proteins as major surface proteins (MSPs), outer membrane proteins (OMPs) and several type 4 secretion system (TFSS) proteins (103) were intensively explored. Those proteins are involved in different function as adhesins to red blood cells and tick epithelial cells (MSP) (104), Adhesin/invasion (OMPs) (105) proteins can mediate transfer of DNA and proteins into eukaryotic host cells, may interfere and are important for the survival of intracellular bacteria (TFSS) (106, 107). The antigens were tested as vaccine candidates in a form of recombinant protein(s), DNA, plasmids, or synthetic peptides and even in some cases, as mixtures of different forms. Recently “quantum vaccinomics” was applied to identify and characterize *A. phagocytophilum* MSP4 protective epitopes by a microarray epitope mapping. The identified candidate protective epitopes, or immunological quantum, were used to design and produce a chimeric protective antigen that was used in vaccination trials of rabbits and sheep. The resulting antibodies from the two types of immunized animal hosts were equally effective to block cell infections in an *in vitro* inhibition assay of *A. phagocytophilum*. The results from these experiments supported the use of quantum vaccinomics as an effective tool for the design of new chimeric candidate protective antigens, as a better alternative to the use of a full-size single protein (MSP4) for vaccine development (108).

#### 3.3.3 Genetically modified *Anaplasma* pathogens

Genetic modification was achieved in *Anaplasma* pathogens either by knocking out biologically important proteins, or by introducing an exogenous gene into its genome to express a protein able to generate protective immune responses. These modifications were performed in *in vitro* culture to achieve genetically modified organisms. A mutant strain was attained in *A. marginale* by transposon mutagenesis of the *A. marginale* Virginia strain to reduce the expression of OMP (109). Although this mutant strain can be transmitted by ticks, it showed reduced infectivity in both intact and splenectomized cattle (110). However, this mutant was not tested for protection against homologous or heterologous challenge. Another immunization trial was performed by the mutant of *A. centrale* with a transposon-mediated insertion of a construct containing Turbo GFP as a marker, and antibiotic resistance genes for selection. Upon animal immunization, it provided immunity and showed clinical signs like the infection with unmodified *A. centrale* but with lower percentage of infected RBCs (110). Both trials were considered as an advance for disease control, but the possibilities of other pathogen transmission like mycoplasmas and even viruses present in the culture systems should also be taken into consideration (92).

No universal vaccine is yet available to protect against diverse geographic strains of *A. phagocytophilum* and *A. platys*, *Anaplasma* species which are known to infect dogs (39) and other animals. *Anaplasma* spp. can be transmitted mainly by *Rhipicephalus* and *Ixodes* for *A. platys* (33, 111) and *Dermacentor, Hyalomma* and *Rhipicephalus* in case of *A. phagocytophilum* (112). Whole genome sequencing of both *A. phagocytophilum* and *A. platys*, allowed for the identification of several potential candidates for vaccine development. It was found that nine of proteins that have an immunogenic potential like the Asp14, Asp55, Msp5, Msp2, AipA, OmpA, APH 0032, and APH 1384 antigens of the type IV secretion system of *A. phagocytophilum* (113–117) can be rational candidates for vaccine development.

### 3.4 *Trypanosoma* parasite vaccines

Currently there is no available vaccine for trypanosomiasis prevention. One of the main challenges to develop a vaccine is the mechanism that *Trypanosoma* parasites use to evade the host immune system, by constantly varying the structure of their surface coating. The antigenic variation operated by the parasites makes it difficult to identify appropriate candidates for vaccine development, especially for the case of subunit vaccines.

There are limited options to prevent trypanosomiasis within vertebrate hosts. In addition, the increase of resistance toward trypanocidal drugs makes chemotherapy, the major means to control infection, difficult to use. Additionally current drugs have various other shortcomings, including toxicity and limited efficacy (118). The drugs commonly used for the treatment of infected domestic animals with *T. evansi* are Diminazene aceturate, which causes some toxicity to the host (119) and isometamidium chloride (120). In this scenario, vector control remains a very important step for disease prevention (121). Another important challenge, despite sporadic reports, is the lack of full knowledge on the geographic distribution of the disease and its transmitted vectors, which affects the control initiatives that require reliable information.

To control this disease, different approaches should be addressed, but the main and more practical strategy is just minimizing contact with the vector tsetse flies. The principal control method is targeting the tsetse fly using insecticides, which has environmental drawbacks. These drawbacks led to the development of bait technologies which include traps and insecticide-impregnated targets (122). Another approach is to use naturally trypano-tolerant breeds of livestock, which is considered as an economical addition to the intervention tools (41).

Previous attempts to develop subunit vaccines against African trypanosome infections have highlighted the difficulties in overcoming the immune evasion mechanisms that have been evolved by these parasites for survival (123). Many antigens were chosen as vaccine candidates. Such is the case of the variable surface glycoprotein (VSG) (124), which provided partial protection, but became non-effective after some time (125). Also, vaccine formulations based on invariant surface glycoproteins and subcellular proteins of the cytoskeleton, like actin and tubulin (123) generated unsatisfactory and non-protective immune responses. However, the advancement in the field of bioinformatics, together with the availability of omics data from different organisms is allowing the design of new generation vaccines that may offer better antigenicity and safety profile. The so-called reverse vaccinology approach, which depends mainly on omics data, permits the design of vaccines that can involve many antigens of different expected important functions. Here are some examples of vaccination trials performed against *Trypanosoma* parasites.

#### 3.4.1 Subunit vaccines against *Trypanosoma* parasites

One vaccine trial was based on a single recombinant protein comprising the extracellular region of a conserved cell-surface protein that is localized to the flagellum membrane invariant flagellum antigen from *T. vivax*. When this protein was used in vaccination in mice it resulted in survival of 10 out of the 15 mice which also were protected beyond at least day 170 (126). This formulation is considered as the first ever successful vaccine trial against this devastating disease caused by *Trypanosoma* parasites (127).

#### 3.4.2 Chimeric vaccine against *Trypanosoma* parasites

Advancements in computational modeling coupled with the availability of large amounts of omics data from different organisms have allowed the design of new generation vaccines. A multi-epitope vaccine (MEV) designed from a collection of antigenic peptides from conserved hypothetical plasma membrane proteins of *Trypanosoma brucei gambiense*. It was expected to give adequate immune stimulation but this vaccine was not tested with parasite challenge (128).

#### 3.4.3 Genetically engineered *Trypanosoma* parasites

Since it is predicted that *T. cruzi* cyclophilin-19 (Cyp19) protein is important for parasite growth, a mutant parasite line lacking the Cyp19 gene was generated. The mutant parasites failed to replicate when inoculated into host cells *in vitro* or in mice, confirming that Cyp19 is critical for infectivity as well. Moreover, immunization with a *T. cruzi* Cyp19 deletion mutant protects 100% upon challenge of the mutant-immunized mice with virulent wild-type parasites, indicating the effectiveness of this line at preventing death from acute disease in mice. In addition, this mutant line did not cause clinical disease in immuno-deficient mice confirming their lack of virulence (129).

#### 3.4.4 mRNA-based *Trypanosoma* vaccines

It is now known that there are many benefits of mRNA-based vaccines, which led researchers to use this approach into vaccine development against *Trypanosoma* parasites. The mRNA-based vaccine approach could prevent disease, but so far, no study was conducted regarding testing this concept (125).

## 4 Vaccines against vectors

### 4.1 Tick vaccines

Tick-borne diseases (TBDs), particularly those caused by blood parasites, pose a significant threat to global livestock industries. The widespread distribution of various tick species, combined with climate change and human activities, contributes to the outgoing expansion of tick populations. Anti-tick vaccines (ATVs) offer a promising, safe, and environmentally friendly approach to tick control. These vaccines not only reduce vector infestations on vertebrate hosts but also hinder the transmission of blood parasites, thereby mitigating the economic impact of tick-borne diseases at both herd and regional levels. ATVs also serve as an effective alternative to chemical tick control, as they are cost-efficient and can be applied across different host species (130). By minimizing the need for acaricides, ATVs help to prevent the emergence of acaricide-resistant tick strains, reduce contamination in the food chain, and promote environmental safety.

Two commercial vaccines were developed based on the glycoprotein Bm86 tick midgut protein, TickGARD Plus (131) and Gavac Plus (132) for use in cattle. This protein was derived from the *Rhipicephalus microplus* ticks, but it is also conserved in other related *Rhipicephalus* ticks. Those vaccines were developed and tested in Australia and Cuba, respectively (133), and were also used in other countries like Mexico and Venezuela against the cattle ticks *R. microplus*, *R. australis* and *R. annulatus* with different levels of efficacy, ranging between 10 and 89% (134, 135). In Cuba and Colombia, those vaccines were generally effective to reduce tick populations and the number of acaricide treatments, in addition to babesiosis and anaplasmosis infections, respectively (136). The TickGARD Plus vaccine is no longer used since 2021 in Australia because of the need for numerous applications (3–4 booster doses per year) (137). So, currently, the only commercially available anti-tick vaccine is Gavac® (138, 139).

Developing effective vaccines against ticks requires intensive research and costly experimental trials. Current approaches in tick vaccine research are focused on identifying potent candidates playing a critical role in tick biology that are capable of inducing cross-reactive immunity against multiple tick species. This requires the identification of highly conserved and functionally relevant antigens that are also exposed to antibodies in the host. It can be anticipated that recent progress in tick omics in addition to other novel molecular biology approaches, and a better understanding of tick biology, will be helpful for the identification of such antigens.

As it was described in the *Babesia* parasite vaccines section above, there is also the hope of developing dual anti-tick and anti-*babesia* vaccines.

### 4.2 Blood sucking flies’ control

To date, there is not a specific vaccine against blood-sucking flies. However, there are several other alternative methods available that can be applied and used in combination to approaches aimed at controlling the blood parasite transmission and its causing diseases, in order to protect livestock. Such methods include the following:

*1-Physical barriers*: Blood-sucking insects can cause allergic reactions in animals, and can also cause different diseases by transmitting several pathogens that are able to propagate in them. Physical barriers can be designed to prevent direct access of flies to animal skin (140). This approach includes the use of protective gear, typically fly sheets, leg wraps and masks. These methods are considered an easy, affordable, and reliable system to prevent insect bites and stings effectively and may prevent the use of chemicals such as insect repellents or insecticides decreasing the environmental hazard.

*2-Insecticides*: Insecticides can be applied to animals and their surroundings either to repel or to kill the blood-sucking flies. Application of insecticides should be used with caution and following the recommended doses to avoid the emergence of insect resistance strains and environmental pollution. Repellents can also be used effectively to prevent flies approaching the skin of target animals. Application of pesticides in animal treatments can be done using automatic sprayers, back rubbers and ear tags (141).

*3-Biological control*: this method, considered as one of the most environmentally friendly approaches, can potentially reduce the current reliance on insecticide-based control. This approach is based on the use of known natural predators or parasites that target blood-sucking flies. Non-insecticide-based strategies have been implemented in different blood sucking as mosquito (142) and ticks by entomopathogenic organisms such as nematodes (143) and protists or mites as biocontrol agents. However, little attention has been given so far to control methods based on biological control, and further exploration studies on the biocontrol of immature and adult stages of blood sucking flies are needed (144).

*4-Environmental management*: This approach involves adequate waste disposal and maintaining clean surroundings, which in turn discourages fly breeding. More importantly, both negative and positive impacts of insects on human and animal health and on the environment need to be addressed by public health professionals. It is also crucial to balance important aspects and goals in insect management approaches, which include regulating their production, exploiting their potential, and limiting their potential negative effect on animals and humans, and on the environment (145).

## 5 Conclusion

The more rational way to avoid blood parasites causing disease is to apply preventive measures against ticks and other transmitting vectors. To have effective and deployable vaccines is not a straightforward effort. There are many obstacles in the way of vaccine development, including the identification of proper vaccine antigen candidates. However, this task is currently facilitated by the availability of data derived from the application of available omics and other approaches, which can provide information on vaccine-targetable proteins that are exposed to the effectors of the immune system and may play key roles in the pathogens’ biology. Ideally, those antigens, in conjunction with proper adjuvants, should be able to induce desirable protective immune responses in immunized animals. Moreover, other aspects such as practicality of vaccine production, the choice of efficient and safe adjuvants (if needed), feasibility of shipment, shelf life, safety of the vaccine, licensing, and commercialization are also important points that also need to be considered for successful vaccine design.

Recombinant protein production is a technology usually applied to vaccine development. However, confirming that the structure of the recombinant protein faithfully resembles that of the native protein should be considered, so the relevant epitopes, including conformational ones, will be antigenic and reachable by the protective antibodies. With the advancements in bioinformatics, we can now predict quite reliably the presence of T or B cell epitopes, but that does not necessarily mean that these antigens will be immunogenic enough to be immunoreactive in the actual immunization trials. In addition, recent developments in vaccinology imply that researchers should also consider other alternative methods of vaccine delivery that do not require the production of recombinant antigens, including DNA and RNA vaccines.

Although live vaccines with or without the addition of genomic modifications had achieved promising results in several vaccine trials, they should be used with caution to avoid massive outbreaks in non-endemic areas populated with animals that were never exposed to such pathogens before. Also, there are still concerns regarding the application of GMOs in the vaccination field, fearing that genomic modifications may have unforeseeable consequences, either to the environment, in other unrelated commensal organisms, or in the animal genomes, with the subsequent impact on humans as well.

To test vaccine candidates and to get the relevant and valid results, researchers should be cautious with the route of pathogen challenge, either as an inoculum form, or by natural infection through the vector (ex. tick challenge). While the former one is considered as a fast way, the latter one acts in a more gradual way, representing a simulation of natural infection in the field.

Next,-Generation Technologies and Systems Biology is considered a modern molecular toolkit, and it is a leader guide for the future direction in vaccine candidate design. The traditional way to identify a novel antigen(s) is usually restricted to individual studies, an approach that can be considered as slow and limited. Sequencing technologies, bioinformatics, and statistics analysis facilitate the omics for lots of parasites. Omics such as genomics and transcriptomics have facilitated the functional annotation of the genome for many of these parasites, which significantly improved the understanding of the parasite biology, interactions with the host, as well as virulence and host immune response (146). To identify ideal vaccine component peptides or proteins, a comprehensive identification of the entire gene repertoire through genome, transcriptome, and proteome data mining, followed by the analysis of their encoding sequences using bioinformatics tools. The analysis should focus on *in silico* characteristics and assess both intra-species (from at least five genomes) and inter-species (among phylogenetically related organisms) conservation levels. Moreover, cutting-edge technologies such as gene editing by CRISPR-Cas9 have also allowed the discovery and functional characterization of potential novel vaccine antigens (146).

**Figure 2:**
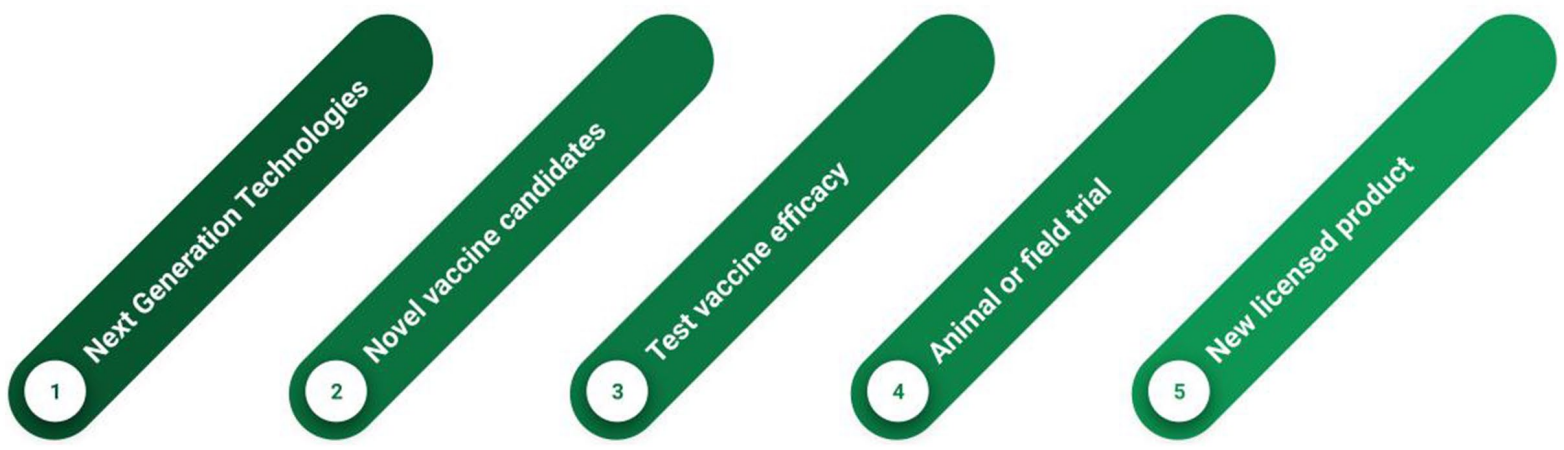
Next-generation technologies leading to novel vaccines pathway.

Finally, even if the vaccine candidates derived from omics technologies, computational approaches, or validated by using genetic manipulation approaches, they still need to be evaluated by clinical or field trial by animal experiments in order to test efficacy and safety. Therefore, systems vaccinology combined with experimental validation and evaluation in animal models through field trials can significantly improve the design of novel vaccines against blood parasites, opening a new era of vaccinology research that could lead to an expansion in licensed products after decades of slow advances (Figure 2).

In the *Babesia bovis* field, there are examples of the use of NGS technologies for the selection of vaccine candidates. The 6cys proteins and Hap2 protein were chosen as transmission blocking vaccine candidates after investigation of the genome annotation since these antigens were known to be involved in sexual stage development in other *Babesia*-related parasites, and then experimentally validated for *Babesia* parasites. Although the recombinant 6Cys proteins used in vaccination trials did not generate an effective transmission blocking response, a vaccine based on recombinant Hap2 was shown to be effective in blocking transmission of *B. bovis* (147), a finding that should be considered for the eventual production of a commercially available TBV against this parasite.

In conclusion, addressing the various challenges in vaccine development is of paramount importance. Recent advances in vaccine technology offer significant opportunities, particularly using multicomponent formulations that incorporate multiple antigens. Utilizing live or whole-cell immunogens, along with a combination of whole attenuated parasites and recombinant sexual stage antigens, generally enhances the effectiveness of vaccines in controlling blood parasite infections and their transmission by vectors. However, it is essential to proceed with caution. The inclusion of a greater number of antigenic subunits may lead to unfavorable three-dimensional structures, which could result in the loss of critical conformational epitopes. Therefore, while the multicomponent approach shows promise, careful attention must be paid to maintaining the structural integrity and immunogenicity of the antigens to ensure the development of an effective vaccine.

## 6 Recommendations

- Based on previous experiences, ideally, future research directions toward development of effective vaccines should involve the prevention of the acute disease, as well as prevention of parasite transmission.
- Sequence variations in vaccine target genes may result in vaccine failures. In order to achieve the goal to control blood parasites and its associated vectors using recombinant proteins, it is important to verify sequence conservation of the target antigens among distinct geographic isolates of the parasites, using, for instance, available sequence databases.
- It is likely that using a single antigen as a vaccine is disadvantageous to induce full protective immune responses. Thus, using multiple antigens as a multicomponent formulation of vital functions or using the live or whole-cell immunogens or a consolidation of the whole attenuated parasite in addition to recombinant sexual stage antigens, would be, in general, more effective vaccine to control blood parasites infection and its transmission by vectors.
- Finally, to ensure disease control and to reduce disease impact, it’s critical to have environmentally friendly vector control measures applied in addition to the use of safe vaccines, in the vector specific season.

## Footnotes

^1^https://www.prnewswire.com/news/university-products-llc/
